# Structural, Optical, Magnetic and Electrochemical Properties of CeXO_2_ (X: Fe, and Mn) Nanoparticles

**DOI:** 10.3390/ma16062290

**Published:** 2023-03-13

**Authors:** Shalendra Kumar, Faheem Ahmed, Nagih M. Shaalan, Nishat Arshi, Saurabh Dalela, Keun H. Chae

**Affiliations:** 1 Department of Physics, College of Science, King Faisal University, P.O. Box 400, Hofuf 31982, Al-Ahsa, Saudi Arabia; 2 Department of Physics, University of Petroleum & Energy Studies, Dehradun 248007, India; 3 Physics Department, Faculty of Science, Assiut University, Assiut 71516, Egypt; 4 Department of Basic Sciences, Preparatory Year Deanship, King Faisal University, P.O. Box 400, Hofuf 31982, Al-Ahsa, Saudi Arabia; 5 Department of Pure & Applied Physics, University of Kota, Kota 324005, India; 6 Advanced Analysis Center, Korea Institute of Science and Technology, Seoul 136-791, Republic of Korea

**Keywords:** CeO_2_, XRD, UV-vis spectroscopy, ferromagnetism, NEXAFS, supercapacitors

## Abstract

CeXO_2_ (X: Fe, Mn) nanoparticles, synthesized using the coprecipitation route, were investigated for their structural, morphological, magnetic, and electrochemical properties using X-ray diffraction (XRD), field emission transmission electron microscopy (FE-TEM), dc magnetization, and cyclic voltammetry methods. The single-phase formation of CeO_2_ nanoparticles with FCC fluorite structure was confirmed by the Rietveld refinement, indicating the successful incorporation of Fe and Mn in the CeO_2_ matrix with the reduced dimensions and band gap values. The Raman analysis supported the lowest band gap of Fe-doped CeO_2_ on account of oxygen non-stoichiometry. The samples exhibited weak room temperature ferromagnetism, which was found to be enhanced in the Fe doped CeO_2_. The NEXAFS analysis supported the results by revealing the oxidation state of Fe to be Fe^2+^/Fe^3+^ in Fe-doped CeO_2_ nanoparticles. Further, the room temperature electrochemical performance of CeXO_2_ (X: Fe, Mn) nanoparticles was measured with a scan rate of 10 mV s^−1^ using 1 M KCL electrolyte, which showed that the Ce_0.95_Fe_0.05_O_2_ electrode revealed excellent performance with a specific capacitance of 945 Fּ·g^−1^ for the application in energy storage devices.

## 1. Introduction

Supercapacitors have gained enormous attention due to their highly demanding applications in sustainable energy storage and harvesting [1,2,3]. Therefore, the development of supercapacitors with enhanced performance is one of the main current research areas. Scientists are widely working in search of new materials useful for the fabrication of important components of supercapacitors, such as electrodes. The key points to enhancing the performance of the supercapacitor are to obtain high energy as well as high power densities and a durable life with stable cycles. Ceria (CeO_2_) is one of the most extensively probed potential candidates due to its fundamental chemical and physical properties [4,5]. However, the properties of CeO_2_ can be easily tailored simply by adding suitable dopant ions into the host lattice for various technological applications such as spintronics [6,7,8,9], electrode materials for supercapacitors [10,11,12], a solid oxide fuel cell [13], and antimicrobial agents [4]. From this perspective, cerium oxide (CeO_2_) appears to be a potential candidate due to its redox properties, which are associated with the reversible oxidation states of cerium (Ce^3+^/Ce^4+^). Reportedly, Rodrigues et al. have investigated the electrochemical performance of CeO_2,_ proving it a reversible redox electrochemical system due to the coexistence of Ce^4+^/Ce^3+^ states [14]. Other than dynamic electrochemistry, nanoceria has additional advantages, such as a high surface-to-volume ratio, oxygen vacancies, and a tunable morphology. The oxygen non-stoichiometry gives rise to certain ionic defects and vacancies, which significantly affect the electronic properties. The oxygen vacancies can modify the conductivity via charge delocalization. Owing to its unique modifiable electronic properties, CeO_2_ takes part in the electrochemical reactions in such a way as to facilitate the charge transport in electronic components [15,16,17,18,19,20,21,22,23,24,25]. In addition, CeO_2_ can display synergistic effects when combined with other materials. Thereby, electrochemically active CeO_2_ may be a suitable electrode material for the development of enhanced-performance supercapacitors. However, despite its various advantages, CeO_2_ has a drawback that restrains its usage in energy storage devices. CeO_2_ exhibits low electrical conductivity [26].

Additionally, the electrochemical properties and charge transport of CeO_2_ based electrodes can be positively modified by doping with various transition metals. The addition of a small fraction of transition metal ions in the CeO_2_ matrix has been found to modify various properties, including the electrochemical activity of the resulting material [7,27,28]. The transition metal doping reduces the crystallite size in CeO_2,_ resulting in an enhancement of the surface-to-volume ratio. Alongside, doping induces oxygen vacancies, which show charge interplay around them, causing an enhancement in charge transport. Moreover, the nature of transition metal ions greatly affects the morphology and electronic structure of the material [19]. The specific capacitance of pure CeO_2_ has been reported to be 235 F/g, which increased after doping with Co and Ni to be in the range ~ 500–600 F/g [14,29]. Similarly, Zr-doped CeO_2_ also showed a specific capacity of 448.1 F/g at a current density of 1 A/g [30]. The device assembled using Zr-doped CeO_2_ displayed capacity retention of 96.4% at larger cycles along with cyclic stability [31]. In addition to the nature of the dopant ion, the synthesis method employed to prepare the material is equally significant. For instance, Prasanna et al. used the combustion method, which favors electrochemical activity by inducing anionic vacancies, to prepare porous nanoceria [32]. The resulting product was found to have created oxygen vacancies on the surface of nanostructures analyzed by x-ray photoelectron spectroscopy. The induced oxygen vacancies, along with the high surface area and adequate pore size, magnified the rate of ion diffusion, due to which the material exhibited a specific capacitance of up to 134.6 F/g at 5 A/g with a retention ratio of up to 92.5%. Likewise, Jeyaranjan et al. have investigated the electrochemical properties of various morphologies (nanoparticles, nanorods, and nanocubes) of nanoceria prepared using the hydrothermal method and reported a specific capacitance of 162.47 F/g for nanorods [33]. It is perceivable that the chemical route methods are more favorable to facilitate the electrochemical reaction by modifying the electronic structure of the material. Herein, the structural, optical, magnetic, electronic structural, and electrochemical properties of CeXO_2_ (X: Fe, Mn) nanoparticles have been reported. 

## 2. Experimental

The undoped and CeXO_2_ (X: Fe, Mn) nanoparticles have been synthesized via the co-precipitation route using the following raw materials: cerium (III) nitrate hexahydrate (432.22 g/mol); manganese (II) nitrate hydrate (178.95 g/mol); iron (II) nitrate nonahydrate (404.0 g/mol); and NH_4_OH solution of highest purity (99.99%) of CDH. The cerium nitrate hexahydrate was used to synthesize the undoped CeO_2_, while the iron nitrate nonahydrate and manganese nitrate hydrate were used for doping along with the cerium nitrate hexahydrate. The precursors were weighed in stoichiometric amounts and added to the deionized water to make 0.06 M of the solution with continuous stirring. The dropwise ammonia solution was added until the pH of the solution reached 9 and was maintained throughout the experiment. After 2.5 h, the stirring was stopped and the solution was centrifuged to get the precipitate. The precipitates were washed with deionized water and ethanol many times to wash out the impurities and then dried in a hot air oven at 80 °C for 24 h. After grinding, the fine powders were sintered at 500 °C for 5 h. The product was finally ground to characterize for various measurements, viz., XRD, TEM, UV-vis spectroscopy, dc-magnetization, and electrochemical analysis.

### Characterization Techniques

A Philips X-pert X-ray diffractometer was used to record the diffraction patterns using Cu Kα (λ ~ 1.5418 Å). The FE-TEM (JEOL/JEM-2100F version) operated at 200 kV and was used to capture the TEM micrographs and selected area electron diffraction (SAED) pattern. The UV-vis spectroscopy measurements were obtained using Model LAMBDA 35, PerkinElmer (Waltham, MA, USA), and Raman spectra were recorded using a Raman spectrometer (NRS-3100) of SINCO Instrument Co. The magnetic behavior of the samples was studied using the quantum design physical properties measurement setup (PPMS). The Corrtest-CS150 workstation was utilized for the electrochemical measurements of CeO_2_, Ce_0.95_Fe_0.05_O_2_, and Ce_0.95_Mn_0.05_O_2_ nanoparticles. All the electrochemical characterizations were performed with a 1 M aqueous solution of KCL as an electrolyte in a conventional three-electrode cell configuration. The working electrodes were designed by mixing 80% active materials (CeO_2_, Ce_0.95_Fe_0.05_O_2_, and Ce_0.95_Mn_0.05_O_2_ nanoparticles), 10% carbon black, and 10% polyvinylidene fluoride (PVDF). The weighted electrode materials were homogeneously mixed using n-methyl-2 pyrrolidinone (NMP) as a solvent to form a slurry. The slurry was pasted on the nickel foam substrate (~1.0 cm^2^) and then dried at 80 °C in the hot air oven for 12 h. All the electrodes were identical with respect to shape and size. The Ag/AgCl and Pt wires were utilized as reference and counter electrodes, respectively. 

## 3. Results and Discussion

### 3.1. XRD Analysis

**Figure 1:**
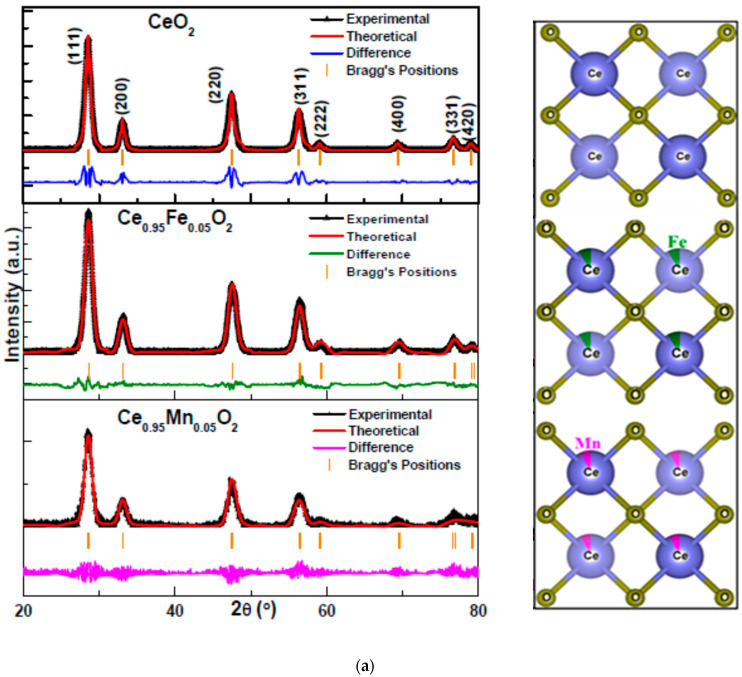

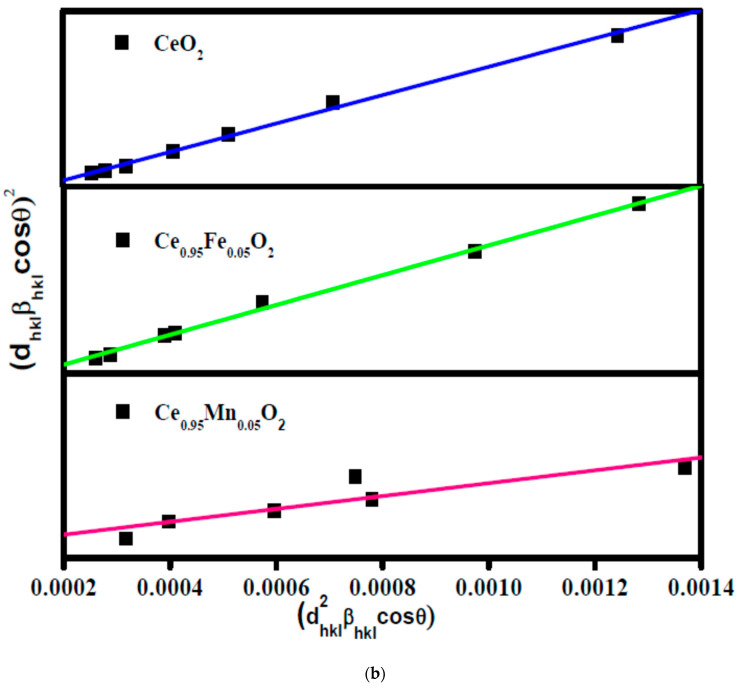
(**a**). Rietveld refined X-ray diffraction patterns of undoped and CeXO_2_ along with the respective images showing incorporation of Fe and Mn in CeO_2_ matrix: Experimental data points are shown in colour, the red line shows superimposed theoretically calculated curve, vertical orange colour lines show Bragg’s positions and blue/green/pink line at the bottom indicate the difference in respective fittings. (**b**) Size-strain plots of undoped and CeXO_2_ (X: Fe, Mn) nanoparticles.

The structural features of the nanoparticles have been investigated using X-ray diffraction patterns. Rietveld refinement of the patterns, performed using open-access Fullprof software, is displayed in Figure 1a. The lattice parameters, peak shape parameters, background, atomic positions, and occupancies are carefully refined using the pseudo-Voigt function. The experimentally observed and theoretically calculated patterns are represented in black and red, respectively, whereas the difference between the two is represented at the bottom by blue, green, and pink colored lines for undoped, Fe-doped, and Mn-doped CeO_2_ nanoparticles, respectively. Bragg’s positions are shown by the vertical orange-colored lines. The values of reliability factors and *χ*^2^ obtained after refinement are mentioned in Table 1. The value of *χ*^2^ is between 1.3–1.5, which is acceptable for the good quality of refinement. The refined crystal structure showing 4 oxygen atoms bonded to 1 Ce atom and the 5% fraction of dopant ions substituted in Ce are displayed along with the respective refined XRD spectra. The indexing of the peaks associates the peak positions to the face-centered cubic fluorite structure (space group: Fm3m) of CeO_2,_ which corresponds to the JCPDS number: 75–0158 [34]. The indexing shows that all the samples attain similar crystallite structures, indicating effective incorporation of Fe^2+^ and Mn^2+^ in place of Ce^4+^. However, the strain is found to be increased in the doped compounds, giving a maximum value for Fe-CeO_2_. As a consequence, the size-strain plot (SSP) calculation is carried out using equation [35], as shown below.

(1) $${\left(d_{hkl}\beta_{hkl}\cos\theta \right)}^{2}=\left(\frac{k}{D}\right)\;d_{hkl}^{2}\;\beta_{hkl}\cos\theta +{\left(\frac{\varepsilon }{2}\right)}^{2}$$

**Figure 2:**
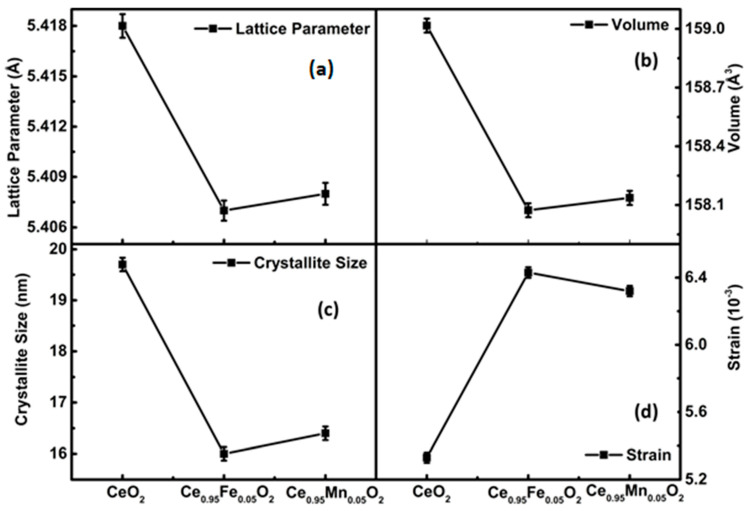
The variation of (**a**) lattice parameter; (**b**) unit cell volume; (**c**) crystallite size and (**d**) strain obtained for undoped and CeXO_2_ (X: Fe, Mn) nanoparticles.

Now, using the aforementioned equation, a plot is made with each diffraction peak’s associated (d^2^_hkl_β_hkl_ cosθ) term along the X-axis and (d_hkl_β_hkl_cosθ)^2^ along the Y-axis, as shown in Figure 1b. The intercept of the straight line provides the intrinsic strain of the nanoparticles, and the slope gives the average size. We observed that the size of the crystallites in all samples calculated using the size strain plot is comparable to that determined using the Scherrer method [5]. Rietveld refinement further reveals a shifting in the peak position towards a higher 2θ value, which indicates the decrease in the lattice parameter as displayed in Figure 2a. Comparing the XRD diffraction peaks (111) of undoped CeO_2_ and Fe- and Mn-doped CeO_2_ nanoparticles, it is evident that the replacement of some Ce^4+^(0.097 nm) ions by smaller radius Fe^2+^(0.077 nm) ions and Mn^2+^(0.087 nm) ions results in an increase in FWHM with a decrease in the crystallite size of the particles. The crystallite structure formed with the smallest unit cell volume is in the case of Fe-CeO_2_ nanoparticles, as displayed in Figure 2b. This also attains the smallest density of the compound, 6.9 g/cm^3^ (see Table 1). Further, Scherrer’s formula is used to calculate the crystallite sizes, which are shown in Figure 2c.

### 3.2. TEM Analysis

**Figure 3:**
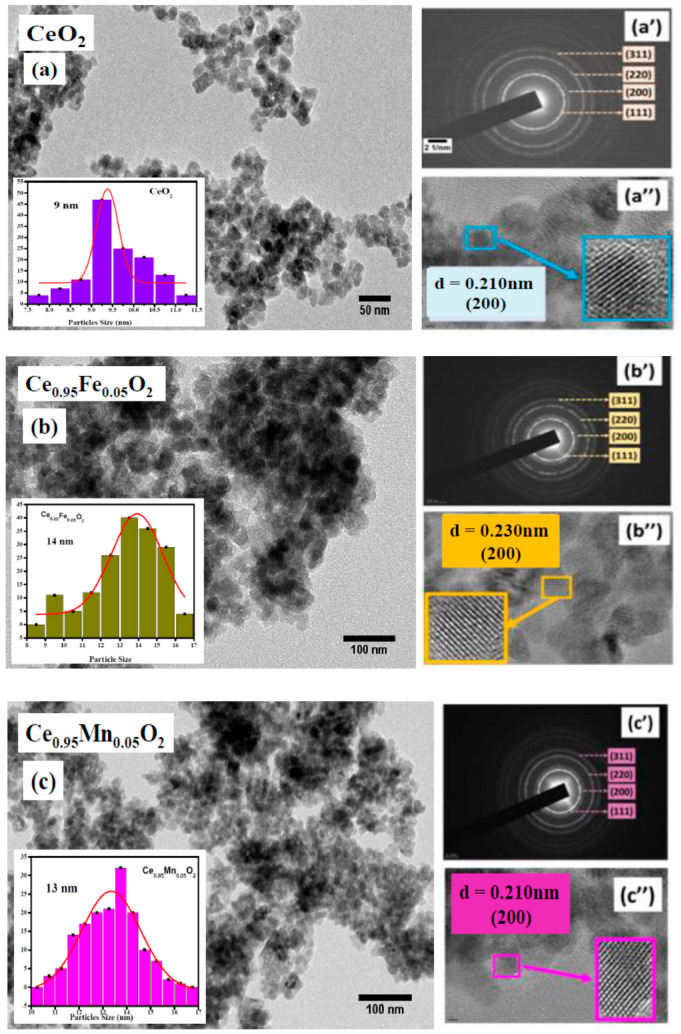
(**a**–**c**) Transmission Electron Micrographs of undoped and CeXO_2_ (X: Fe, Mn) nanoparticles; inset shows the histograms representing particle size distribution fitted with Gauss function; (**a′**–**c′**) shows the SAED patterns and (**a″**–**c″**) highlights the HRTEM patterns of CeXO_2_ (X: Fe, Mn) nanoparticles.

The morphology of the nanoparticles is analyzed through TEM micrographs, as demonstrated in Figure 3a–c. The spherical particles with aggregation can be seen in the micrographs with unaffected morphology for Fe^3+^ and Mn^2+^ion incorporation in the CeO_2_ nanolattice. The particle sizes are calculated using open-access ImageJ software and are represented through the fitted size distribution histograms shown in the insets of respective Figure 3a–c. The histograms show a narrow particle size distribution, indicating uniformity in the particle sizes. Mn-CeO_2_ nanoparticles have the smallest particle size of 13 nm as compared to CeO_2_. The indexing through selected area electron diffraction (SAED) ring patterns, shown in Figure 3a′–c′ confirms the face-centered cubic fluorite structure of all the samples. The indexed planes are marked along with the ring patterns and indicate small-sized particles with increased crystallinity for Fe and Mn cation doping in the CeO_2_ nanolattice. Further, the high-resolution transmission electron micrographs are displayed in Figure 3a″–c″. The interplanar spacing for undoped CeO_2_, Fe-, and Mn-doped CeO_2_ nanoparticles is approximately 0.210, 0.230, and 0.210 nm, respectively, corresponding to the (200) plane of fluorite structure.

### 3.3. UV-Vis Absorption Spectra

**Figure 4:**
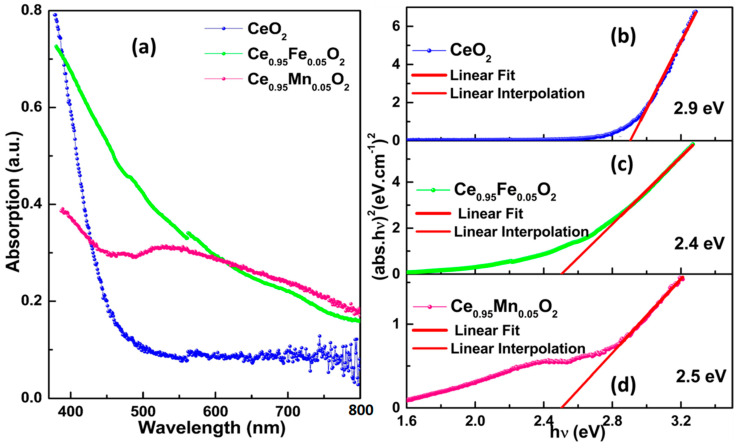
(**a**) UV-vis absorption curves for undoped and CeXO_2_ (X: Fe, Mn) nanoparticles; (**b**–**d**) Tauc’s plots to determine the band gap of the respective samples.

Figure 4a displays the absorption spectra in the wavelength range of 400–800 nm. The spectra show the maximum absorption at 400 nm, which decreases with the increase in wavelength. The highest absorption has been obtained for undoped CeO_2_. The electronic band gap of nanoparticles is calculated with Tauc’s plots, which are displayed in Figure 4b–d for undoped CeO_2_, Fe-CeO_2,_ and Mn-CeO_2,_ respectively. The band gap energy of undoped CeO_2_ nanomaterials (2.9 eV) is smaller in comparison to its bulk counterpart, CeO_2_ (3.35 eV), which may be the outcome of a shift in the 4f electronic states from Ce^4+^ (4f^0^) to Ce^3+^ (4f^1^), which indicates the introduction of an extra electron in the 4f orbital and decreases the band gap energy of undoped CeO_2_ nanomaterials. The highest band gap is found to be 2.9 eV for the undoped CeO_2,_ while the lowest band gap (eV) is exhibited by Fe-doped CeO_2_ nanoparticles. The lowering of the band gap may be associated with the smallest ionic radii and oxidation state of Fe ions. When Fe^2+^ is doped in the CeO_2_ lattice, it substitutes in place of the host cation, Ce^3+^. There is a difference between the oxidation states and ionic radii of Fe ions and Ce ions that leads to the creation of the defect states in the lattice. These defect states are very likely to be the oxygen vacancies in such cases of diluted magnetic semiconductors. These oxygen vacancies create intermediate states via exchange interactions with neighboring electrons, which reduces the band gap of the material. Therefore, the decrease in band gap energy in our doped nanoparticles may be induced by the development of localized impurity defect levels brought on by Fe^2+^ and Mn^2+^ ion doping, which manifests in oxygen vacancies and Ce^3+^ defects.

### 3.4. Raman Spectroscopy

**Figure 5:**
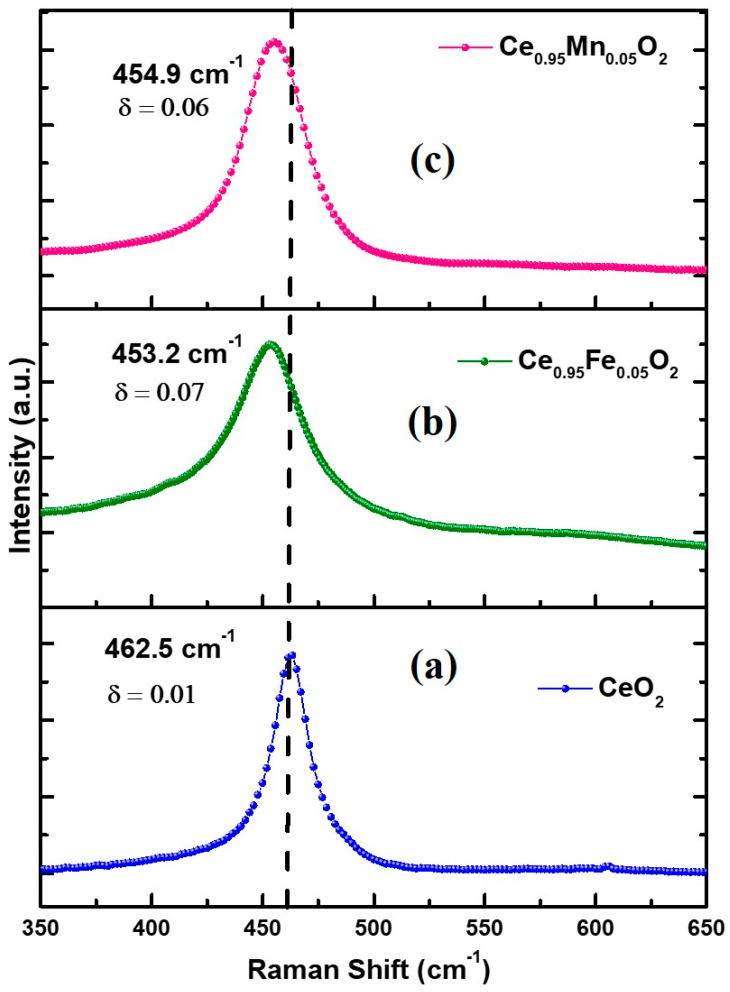
(**a**–**c**) Raman spectra of undoped and CeXO_2_ (X: Fe, Mn) nanoparticles.

The influence of the dopant ion is investigated using the molecular vibrations of the Ce-O8 vibrational unit of the CeO_2_ matrix [36]. The substitution of TM in place of Ce affects the symmetrical stretching of O-ions around Ce-ions, and the resulting vibrations have been detected through Raman spectra, as represented in Figure 5a–c. The spectra show the F_2g_ Raman active modes corresponding to CeO_2_, which are sensitive to the molecular disorder around Ce ions. The characteristic symmetrical breathing Raman active mode F_2g_ of cubic fluorite CeO_2_ is obtained at ~460 cm^−1^, which corresponds to the oxygen ions around Ce^4+^ ions (O-Ce-O) [37]. This Raman peak for undoped CeO_2_ nanomaterials is caused by the growth of oxygen vacancies at the Ce^3+^ site as a consequence of the change of the valence state of Ce^4+^ ions to Ce^3+^ ions. In the present case, the bands are obtained at ~462 cm^−1^, 453 cm^−1^, and 455 cm^−1^ for undoped, Fe doped, and Mn-doped CeO_2_, respectively, which are closer to the characteristic band indicative of the effective substitution of dopant ions in place of the host cation. However, a decrease in the Raman frequency is clearly observed, indicating the occurrence of oxygen non-stoichiometry with O/Ce < 2. The reduction in oxygen content has been found by calculating the value of oxygen deficit (δ) using the formula: δ = 2.66 (1 − ω_n_/ω_b_), where ω_n_ is the position of the Raman active bands of the samples and ω_b_ is the frequency of the bulk CeO_2_ (465 cm^−1^) [29]. The values of δ are indicated in the respective Figure 5a–c, which reveal that even undoped CeO_2_ shows a slight oxygen deficiency. It is noteworthy here that when the particle size reduces from bulk to nm scale, noticeable changes take place in the host lattice, including size confinement effects that may lead to oxygen non-stoichiometry. Further, the substitution of Fe and Mn in place of Ce creates more oxygen non-stoichiometry. The introduction of oxygen vacancies in the CeO_2_ nanolattice leads to a change in the oxidation states of Ce from +4 to +3, which is favorable for the redox properties and therefore influences the density of states, which further affects the important properties of the material.

### 3.5. Magnetisation 

**Figure 6:**
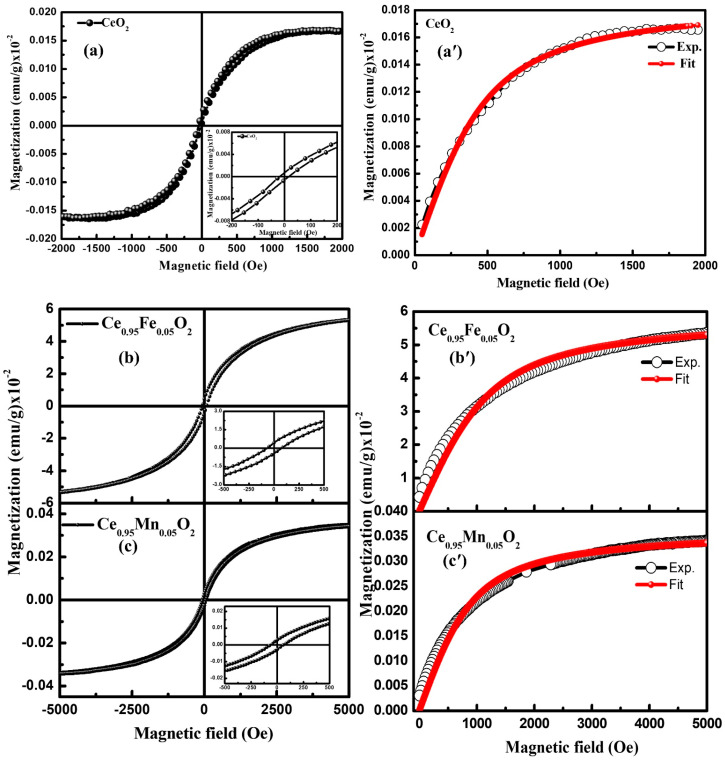
(**a**–**c**) MH hysteresis loops for undoped CeO_2_ and Ce_0.95_X_0.05_O_2_ (X: Fe, Mn) nanoparticles; (**a′**–**c′**) Fitting of M-H loops using Langevin function to find BMP and matrix contribution to the ferromagnetic behavior.

The magnetization behavior of CeO_2_, Fe-CeO_2_, and Mn-CeO_2_ nanoparticles has been studied using VSM at room temperature. The hysteresis loops showing magnetization versus magnetic field (M-H) are displayed in Figure 6a–c. The respective insets show the M-H curve at a low field and infer that all the samples demonstrate weak ferromagnetic ordering at room temperature. The various magnetic parameters such as saturation magnetization (M_S_), remnant magnetization (M_R_), and coercivity (H_C_) are calculated for the undoped and X (Fe, Mn) doped CeO_2_ nanoparticles (see Table 2). The undoped CeO_2_ has the lowest Ms value ~1.5 × 10^−4^ emu/g which changes after doping and shows the maximum value for Fe-doped CeO_2_ nanoparticles. Although there have been numerous theoretical and experimental investigations on RTFM in these oxides [7,8], there is still great controversy regarding the ferromagnetic ordering in these oxides with rare earth and transition metal cation doping and its correlation to the formation of defects and oxygen vacancies. The main condition in CeO_2_ for the observation of ferromagnetic behavior is its tendency towards oxygen non-stoichiometry. When transition metal ions are doped in the CeO_2_ lattice, they create defects such as oxygen vacancies, which interact with the neighboring electron [38]. The oxygen vacancies entrap the electrons, which undergo exchange interactions and create bound magnetic polarons (BMP), which induce ferromagnetic ordering [39].

In order to get more insights into the contribution of BMP to the ferromagnetic behavior of the samples, the M-H loops are fitted with the Langevin function (L(x)). The Langevin function has been employed as described in the literature [40,41,42,43]. The expression may be written as M = M_o_ L(x) + χmH and is simplified as M = A[coth(B∙H)-(B∙H)^−1^] + C∙H, where A = M_o_ = N∙m_s_ (N being the number of BMPs per cm^3^), B = m_eff_/K_B_T (K_B_ being the Boltzmann constant and T being the temperature of taking measurements), and C = χ_m_ (χ_m_∙H is the matrix contribution). The m_s_ (~m_eff_) is the true spontaneous magnetic moment. The values obtained for these parameters are indicated in Table 2. The value of M_o_ is found to be highest for the Ce_0.95_Fe_0.05_O_2_ and lowest for pure CeO_2_, even though the true spontaneous magnetization (m_eff_) is observed to be in reverse order. A similar case has been reported by Mohanty et al., indicating m_eff_ varying inversely from the spontaneous magnetization, which has been attributed to the competing exchange interactions between BMPs and the matrix [43]. Further, the values of N and χ_m_ are also found to be highest for Ce_0.95_Fe_0.05_O_2_. Thus, the values of M_o_, N, and χ_m_ are observed to be lowest for pure CeO_2_ and highest for Ce_0.95_Fe_0.05_O_2_, indicating enhanced ferromagnetic behavior in Ce_0.95_Fe_0.05_O_2_, which confirms the formation of BMPs as a consequence of doping as well as the contribution of the matrix. Since the dopant concentration is the same (5%) in both the doped samples, the enhanced ferromagnetic ordering can not only be associated with the dopant concentration; however, the nature of the elements, i.e., Fe and Mn, also plays a part. Although individual Mn atoms have a higher magnetic moment than Fe, Mn is likely to dwell in the matrix antiferromagnetically, which may be the possible reason for the higher magnetic behavior induced in Fe doped CeO_2_ as compared to Mn doped CeO_2_. In addition, the oxidation state may significantly affect the exchange interactions as Mn is possibly incorporated in the host matrix in the Mn^2+^ oxidation state (see Section 3.6) and Fe is in Fe^2+^/Fe^3+^ mixed valence states, which leaves Fe with more electrons and/or induces a higher number of oxygen vacancies in the matrix. Thus, the doping of Fe in the CeO_2_ matrix enhances the ferromagnetic behavior of the host system.

### 3.6. Near Edge X-ray Absorption Fine Spectroscopy (NEXAFS)

**Figure 7:**
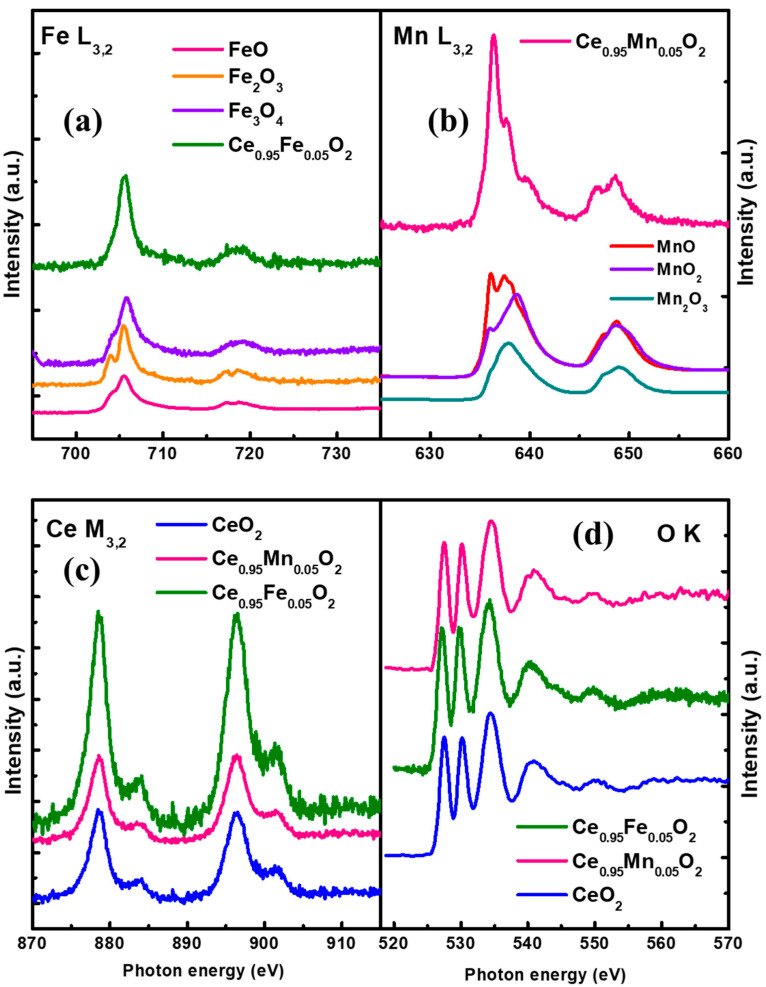
NEXAFS spectra of (**a**) Fe L_3,2_ edge of Ce_0.95_Fe_0.05_O_2_ nanoparticles; (**b**) Mn L_3,2_ edge Ce_0.95_Mn_0.05_O_2_ nanoparticles; (**c**) Ce M_5,4_ edge undoped and CeXO_2_ (X: Fe, Mn) nanoparticles, and (**d**) O K edge of undoped and CeXO_2_ (X: Fe, Mn) nanoparticles.

The oxidation states of the ions are investigated using the NEXAFS spectra shown in Figure 7a–d. Figure 7a displays the L_3,2_ edge spectra of Fe-doped CeO_2_ at ~ 705 and 709 eV together with the reference spectra of FeO, Fe_2_O_3_, and Fe_3_O_4_. The L_3,2_ spectra arise due to the electronic transitions between the core levels of Fe 2p_3/2_ and 2p_1/2_ states and outer Fe 3d states. These transitions result in the formation of holes that take part in charge transfer processes [14]. The L_3_ edge of Fe-doped CeO_2_ shows no splitting and shows more resemblance to that of Fe_3_O_4_, indicating that Fe is dissolved in mixed valence states (Fe^2+^/Fe^3+^). Similarly, Figure 7b displayed the L_3,2_ edge spectra of Mn-doped CeO_2_ along with the reference spectra of MnO, MnO_2_, and Mn_2_O_3_. The spectra of Mn-doped CeO_2_ resemble more closely those of MnO, which show the presence of Mn^2+^ states in the host matrix. Thus, the various enhanced properties in Fe-doped CeO_2_ can be associated with the mixed valence states of Fe, which are responsible for the reduced oxygen stoichiometry in the lattice and the formation of oxygen vacancies. We conclude that the electronic structure of CeO_2_ changes due to a change in the vacant number of 4f orbitals and hybridization with the lattice oxygen, although TM does not show any secondary phases in the CeO_2_ lattice.

Figure 7c depicts the normalized M_5,4_ edge XAS spectra of CeO_2_ and TM-doped CeO_2_. The two white lines at 878 eV and 896 eV on the M_5,4_-edge XAS of CeO_2_ correspond to the electron transitions Ce 3d_5/2_→4f_7/2_ (M_5_) and Ce 3d_3/2_→4f_5/2_ (M_4_) [44]. The spectral white lines M_5_ and M_4_ are approximately 17.91 eV apart. Furthermore, due to changes in spectral characteristics caused by Fe and Mn doping in CeO_2_, the position of the Ce M_5,4_-edges has been seen to move somewhat towards lower energy. The post-edge two satellites appear due to the transition of the 4f state in the conduction band; therefore, these peaks are the distinctive peaks to show the contribution of 4f^o^ states, and the strength of the satellite peaks is used to measure the quantity of the 4f^o^ state. When TM is introduced, it forms oxygen vacancies and increases the fraction of Ce^3+^ in comparison to undoped CeO_2_. Figure 7d represents the O K edge spectra of the samples, indicating transitions from O 1s to hybridized higher energy states. 

### 3.7. Electrochemical Study

**Figure 8:**
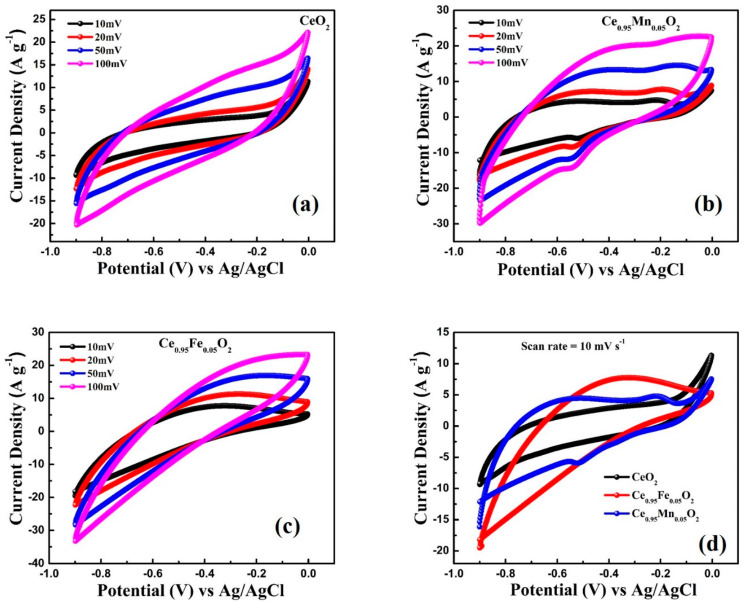
CV plots of (**a**) CeO_2_ nanoparticles, (**b**) Ce_0.95_Mn_0.05_O_2_ nanoparticles, (**c**) Ce_0.95_Fe_0.05_O_2_ nanoparticles with different scan rates (**d**) CV plots CeO_2_, Ce_0.95_Mn_0.05_O_2_, Ce_0.95_Fe_0.05_O_2_ nanoparticles at a scan rate of 10 mV S^−1^.

**Figure 9:**
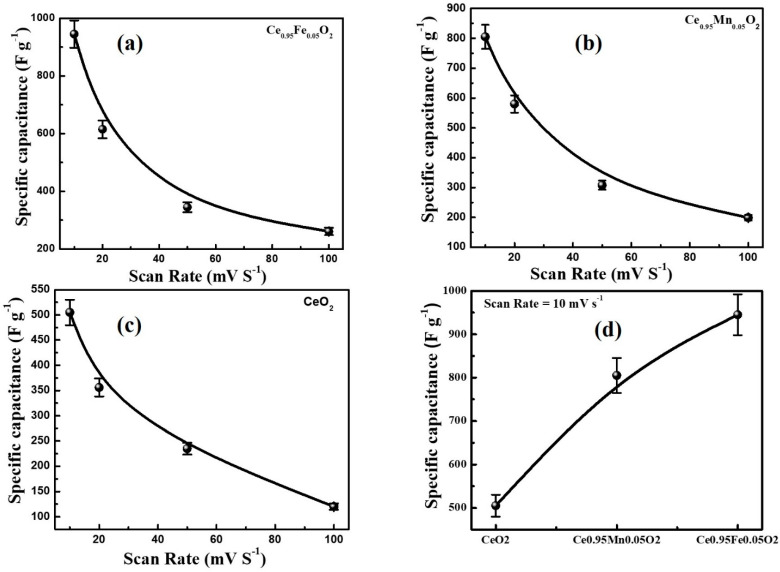
The specific capacitance of (**a**) CeO_2_ nanoparticles, (**b**) Ce_0.95_Mn_0.05_O_2_ nanoparticles, (**c**) Ce_0.95_Fe_0.05_O_2_ nanoparticles with different scan rate (**d**) Variation in specific capacitance of CeO_2_, Ce_0.95_Mn_0.05_O_2_, Ce_0.95_Fe_0.05_O_2_ nanoparticles at a scan rate of 10 mV S^−1^.

The CV measurements are performed to study the capacitance performance of CeO_2_, Ce_0.95_Mn_0.05_O_2_, and Ce_0.95_Fe_0.05_O_2_ electrodes using the three-electrode system in 1 M KCL electrolyte. The CV curves of CeO_2_, Ce_0.95_Mn_0.05_O_2_, and Ce_0.95_Fe_0.05_O_2_ nanoparticles, as highlighted in Figure 8a–d, were measured at different potential scan rates of 10, 20, 50, and 100 mV s^−1^. The CV measurement was done in the potential window of −0.9 V and 0.0 V. It is worth noticing that the features of the CV curves for all electrodes are analogous. The comparison of the CV profiles of CeO_2_, Ce_0.95_Mn_0.05_O_2_, and Ce_0.95_Fe_0.05_O_2_ electrodes measured at a scan rate of 10 mV s^−1^ has been displayed in Figure 8d. One can notice from Figure 8d that the Ce_0.95_Fe_0.05_O_2_ electrode has a higher area under the curve compared to other electrodes. The specific capacitance (C_S_) calculated using the CV profiles of the CeO_2_, Ce_0.95_Mn_0.05_O_2_, and Ce_0.95_Fe_0.05_O_2_ electrodes has been shown in Figure 9a–c. The specific capacitance (C_S_) values are determined using the following relation: CS=nAΔV×m×v, where v denotes the scan rates (V/s), m (g) is the mass of the active material deposited on the electrode, $\Delta V\left(V\right)$ represents the potential window, and n = 1 is used for the three-electrode cell. It is observed that the Cs values decrease with an increase in scan rates (10 mV s^−1^–100 mV s^−1^) from 205 F·g^−1^ to 120 F·g^−1^, 805 F·g^−1^ to 199 F·g^−1^, and 945 F·g^−1^ to 261 F·g^−1^ for CeO_2_, Ce_0.95_Mn_0.05_O_2,_ and Ce_0.95_Fe_0.05_O_2_, respectively, which indicates the usual performance of supercapacitors. Figure 9d describes the comparison of the C_s_ for different electrodes and highlights that the Ce_0.95_Fe_0.05_O_2_ electrode has the highest specific capacitance value of 945 F·g^−1^.

## 4. Conclusions

The undoped and Ce_0.95_X_0.05_O_2_ (X: Fe, Mn) nanoparticles, synthesized using the coprecipitation route, are studied for their structural, morphological, optical, magnetic, electronic, and electrochemical properties. The Rietveld refinement has revealed the single-phase formation of the face-centered fluorite structure of CeO_2_. The Fe and Mn have been successfully incorporated into the CeO_2_ matrix. The lattice parameters and crystallite dimensions are found to be lowest for Fe-doped CeO_2_, mainly because of the lowest ionic radii of Fe as compared to Mn and Ce. The reduced dimensions led to the enhanced strain in Fe-doped CeO_2_ nanoparticles. The particle size obtained from the TEM micrographs also favored the XRD results. The band gap is also found to be minimal for Fe-doped CeO_2_ nanoparticles. The Raman spectra revealed the maximum oxygen non-stoichiometry in the Fe-doped CeO_2_ nanoparticles. The ferromagnetism can be seen for all the nanoparticles with a small hysteresis at room temperature. The smallest value of saturation magnetization and the magnetic moment has been found for pure CeO_2_ nanoparticles and is observed to be enhanced as a consequence of doping, with the highest value for Ce_0.95_F_0.05_O_2_ nanoparticles. The presence of oxygen vacancies is confirmed by Raman and NEXAFS analyses, which also exhibit a mixed valence state for Fe-ions (Fe^3+^ and Fe^2+^) and Ce-ions (Ce^3+^ and Ce^4+^). The cyclic voltammetry results demonstrate that the Ce_0.95_F_0.05_O_2_ electrode displayed the maximum value of specific capacitance (945 F g^−1^) recorded at 10 mVs^−1^ scan rate.

## Figures and Tables

**Table 1:** The reliability factors, densities, and occupancies were obtained through Rietveld Refinement of undoped and CeXO_2_ (X: Fe, Mn) nanoparticles. The crystallite sizes and particle sizes were calculated using various methods.

	R_p_ (%)	R_w_p (%)	R_exp_ (%)	χ^2^	Crystallite Size (nm)		Particle Size (nm)	Density ρ (g/cm^3^)	Occupancy	
									Initial	After Refinement
					Scherrer Method	SSP	TEM		Ce/X/O	Ce/X/O
CeO_2_	20.4	26.6	21.6	1.5	8.40	5.13	9	7.361	0.02083/ 0.04167	0.03336/ 0.08165
Ce_0.95_Fe_0.05_O_2_	14.2	10.1	47.2	1.4	7.14	7.37	14	6.901	0.0197885/ 0.001042/ 0.04133	0.01956/ 0.00177/ 0.04148
Ce_0.95_Mn_0.05_O_2_	31.2	40.5	35.4	1.3	6.34	7.05	13	7.350	0.0197885/ 0.001042/ 0.04133	0.01839/ 0.00142/ 0.04133

**Table 2:** List of parameters obtained from experimental M-H curve and fitting of the Langevin equation to find BMP and matrix contribution to the ferromagnetic behavior.

	Experimental Data			Fitting Parameters Extracted from BMP Model			
	M_s_ (emu/g)	M_r_ (emu/g)	H_c_ (Oe)	M_o_ (emu/g)	m_eff_ (μ_B_)	N (cm^−3^)	χ_m_ (emu g Oe^−1^)
CeO_2_	1.5 × 10^−4^	5.7 × 10^−6^	20.0	1.97 × 10^−4^	2.12 × 10^−16^	9.3 × 10^11^	5.8 × 10^−9^
Ce_0.95_Fe_0.05_O_2_	3.5 × 10^−4^	3.2 × 10^−5^	60.0	0.058	0.77 × 10^−16^	7.4 × 10^14^	2.076 × 10^−7^
Ce_0.95_Mn_0.05_O_2_	5.6 × 10^−2^	4.3 × 10^−3^	80.0	3.26 × 10^−4^	1.1 × 10^−16^	3.0 × 10^14^	6.02 × 10^−9^

## Data Availability

Available on request.
